# Functional outcome, cognition and quality of life after out-of-hospital cardiac arrest and therapeutic hypothermia: data from a randomized controlled trial

**DOI:** 10.1186/s13049-014-0084-9

**Published:** 2015-02-06

**Authors:** Marjaana Tiainen, Erja Poutiainen, Tuomas Oksanen, Kirsi-Maija Kaukonen, Ville Pettilä, Markus Skrifvars, Tero Varpula, Maaret Castrén

**Affiliations:** Department of Neurology, Helsinki University Hospital, Haartmaninkatu 4, Helsinki, 00029 Finland; Institute of Behavioral Sciences, University of Helsinki, Helsinki, Finland; Intensive Care Units, Department of Anaesthesiology and Intensive Care Medicine, Helsinki University Hospital, Helsinki, Finland; Karolinska Institutet, Institution of Clinical Science and Education and Stockholm, Stockholm, Sweden; Helsinki University and HUCH Emergency Care, Helsinki University Hospital, Helsinki, Finland

**Keywords:** Cardiac arrest, Neurological outcome, Cognition, Quality of life, Hypothermia

## Abstract

**Background:**

To study functional neurologic and cognitive outcome and health-related quality of life (HRQoL) in a cohort of patients included in a randomised controlled trial on glucose control following out-of-hospital cardiac arrest (OHCA) from ventricular fibrillation (VF) treated with therapeutic hypothermia.

**Methods:**

Patients alive at 6 months after being discharged from the hospital underwent clinical neurological and extensive neuropsychological examinations. Functional outcome was evaluated with the Cerebral Performance Category scale, the modified Rankin scale and the Barthel Index. Cognitive outcome was evaluated by neuropsychological test battery including two measures of each cognitive function: cognitive speed, execution, memory, verbal skills and visuospatial performance. We also assessed quality of life with a HRQoL 15D questionnaire.

**Results:**

Of 90 OHCA-VF patients included in the original trial, 57 were alive at 6 months. Of these, 52 (91%) were functionally independent and 54 (95%) lived at their previous home. Focal neurological deficits were scarce. Intact cognitive performance was observed in 20 (49%), mild to moderate deficits in 14 (34%) and severe cognitive deficits in 7 (17%) of 41 patients assessed by a neuropsychologist. Cognitive impairments were most frequently detected in executive and memory functions. HRQoL of the CA survivors was comparable to that of age- and gender matched population.

**Conclusions:**

Functional outcome six months after OHCA and therapeutic hypothermia was good in the great majority of the survivors, and half of them were cognitively intact. Of note, the HRQoL of CA survivors did not differ from that of age- and gender matched population.

## Background

The prognosis of patients resuscitated from out-of-hospital cardiac arrest (OHCA) with ventricular fibrillation (VF) as the initial rhythm has improved, as up to 55% of hypothermia-treated OHCA-VF patients may achieve good outcome [1-3]. Long-term mortality among patients discharged alive after OHCA does not differ markedly from that of myocardial infarct (MI) patients without OHCA [4]. In a recent study of Lindner and colleagues, the five-year survival rate for OHCA patients discharged from hospital alive was 75%, and the mean potential life-years saved per patient was 22.8 years [5]. However, not only survival but also functional outcome and quality of life are important long-term outcomes.

Neurologic outcome after CA is commonly evaluated by Glasgow-Pittsburgh Cerebral Performance Categories (CPC) [6,7]. This five-step category classification is simple, but it has a limited value in discriminating between mild and moderate brain injury [8]. Cognitive deficits may markedly impair the functional status of CA survivors and their quality of life. Regrettably, the CPC classification does not comprise cognitive impairment in conscious subjects, unless the impairment is severe. The increased survival of OHCA-VF patients in the hypothermia-era does not seem to be associated with decrease in survivors with clinically significant cognitive deficits [9,10]. Comprehensive data on functional outcome of OHCA patients is still limited. As the number of CA survivors is increasing, there is clearly a need for data of their functional outcome and quality of life.

Accordingly, we aimed to evaluate the functional neurologic and cognitive outcome of hypothermia treated OHCA-VF survivors and their quality of life in a cohort of OHCA-VF patients included in a randomised controlled trial on strict versus moderate glucose control.

## Methods

This study protocol was approved by the ethics committee of the Helsinki University Central Hospital (HUCH). All postresuscitation patients in the HUCH area with witnessed OHCA caused by VF and admitted to the two participating intensive care units (ICU) from November 2004 to December 2006 were screened for the SUGAR-trial. The inclusion criteria were VF of presumed cardiac origin, witnessed arrest, age ≥ 18 years, basic life support (BLS) delay less than 15 min, return of spontaneous circulation (ROSC) less than 35 min and unconsciousness at hospital admission. Exclusion criteria were persistent hypotension (mean arterial pressure below 65 mmHg for over 30 min) despite therapy, pregnancy, terminal illness, pre-arrest illness limiting follow-up (eg. dementia), or a do not attempt resuscitation order. After obtaining informed consent from a close relative, patients were randomized into a strict (4–6 mmol/l) or a moderate (6–8 mmol/l) glucose control group for the first 48 hours of treatment in the ICU. Short-acting insulin was used in both groups as needed. All patients received therapeutic hypothermia of 33°C for 24 hours induced with an intravascular cooling device (CoolGard, Zoll Medical Corporation), followed by slow warming (warming rate not exceeding 0.5°C per hour) to normothermia. General treatment of the patients was conducted according to the ICU’s written standard protocols. The delay from discontinuation of sedative medication to recovery of consciousness (defined as ability to obey verbal commands) was recorded. The cause of CA was classified as acute MI, myocardial ischemia without infarction, primary arrhythmia, or other. The short-term outcome of the SUGAR-trial has been published previously [11].

### Evaluation of outcome

All patients alive 6 months after CA were contacted and invited for a follow-up visit. The evaluation at follow-up visit included an interview and standard neurologic examination performed by the same board certified neurologist (MT). For institutionalized patients the assessment included also an interview with the nearest relatives and/or with nursing staff. The neurological outcome was also assessed by modified Rankin Scale (mRs) [12], Barthel Index (BI) [13], and National Institutes of Health stroke scale (NIHSS) [14]. Cognitive outcome was evaluated by neuropsychological examination including two measures of each cognitive function: cognitive speed, execution, memory, verbal skills and visuospatial performance. Health-related quality of life (HRQoL) was assessed by the 15D questionnaire [15]. The neurologist and the neuropsychologist performing the evaluations were unaware of the patient’s glucose treatment group. If a patient was not able or not willing to attend a follow-up visit, a telephone interview was performed, with evaluation of CPC, BI and mRs.

Modified Rankin scale is a widely applied measure of global disability and handicap after stroke [12]. The scores for patients alive range from 0 (no symptoms) to 5 (bedridden, incontinent, and requires constant nursing care and attention). Favourable outcome in stroke studies is defined as mRs 0-2 (0 = no symptoms at all, 1 = no significant disability despite symptoms, 2 = slight disability; independent but unable to carry out all previous activities). The Barthel Index is a measure to assess an individual's ability to perform activities of daily living related to self-care and mobility; for example, transfers, stairs, feeding, dressing, personal care and bathing [13]. The range of functionally independent outcome is 95 to 100. BI score 90-55 indicates moderate dependency, and score 50-0 indicates full dependency. NIHSS is a widely used instrument for the evaluation of neurologic impairment after stroke [14]. A 15-item scale provides a quantitative measure of the key components of a standard neurologic examination, with higher scores indicating greater impairment.

Neuropsychological examination was designed to estimate cognitive functions sensitive to CA related cognitive deficits. Different cognitive functions were measured by the Similarities, Block Design and Digit Symbol subtests of the Wechsler Adult Intelligence Scale-Revised (WAIS-R), and by the Logical Passages subtask of the Wechsler Memory Scale-Revised (WMS-R) and the List Learning task of the WMS-III [15-17]. Furthermore, the Trail-Making Test (Parts A and B), the Interference and naming subtasks of the modified Stroop Test, semantic fluency task (animal names) and visual search task were used [18,19]. A patient’s test performance was categorized as normal or impaired using the cut point of one standard deviation (SD) below the mean of the Finnish normative sample. If among the 10 tests none or only one (≤10%) was impaired a subject’s cognitive performance was considered intact. When two to four tests (11- 49%) were below 1 SD cut point a cognitive functioning was scored as mildly to moderately defective, and when at least half of the tests (≥50%) were impaired it was scored as severely defective.

15D is a generic, standardized, non-disease-specific self-administered multidimensional measure of HRQoL [20]. It has 15 dimensions: mobility, vision, hearing, breathing, sleeping, eating, speech, elimination, usual activities, mental function, discomfort and symptoms, depression, distress, vitality and sexual activity. Each dimension is divided into five grades of severity. The 15D can be used both as a profile and single index score measure. The single index score, 15D score on a 0–1 scale, represents the overall HRQoL, and is calculated from the health state descriptive system by using a set of population-based preference or utility weights. The maximum score is 1 (no problems on any dimensions) and the minimum score 0 (being dead). The minimal clinically significant difference in 15D is 0.03 [20]. The 15D instrument has been tested in various states of illness, e.g. in invasive treatment of coronary artery disease [21] and stroke [22,23].

### Statistical analysis

We present categorical variables as counts and percentages, and non-normally distributed continuous data as median and range, compared with the Mann-Whitney *U*-test. We compared binary outcome data by Fisher’s exact test. Correlations were analyzed by Spearman’s rho-test. Cognitive functions were analyzed using means and standard deviations. P values < 0.05 were considered statistically significant. We used the Statistica data analysis software system® (StatSoft, Tulsa, OK, USA) to analyze the data.

## Results

**Figure 1:**
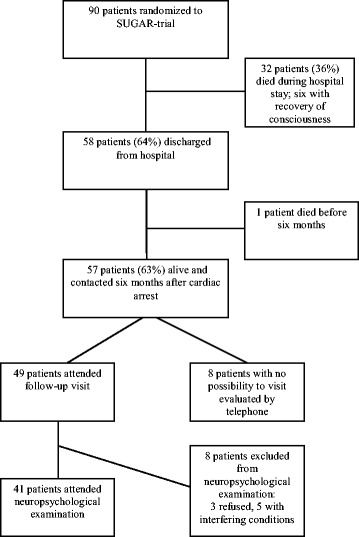
**Flow-chart of study patients.**

At six months after CA, 57 patients (63%) of 90 patients included in the study were alive and were contacted. Outcome was assessed on a follow-up visit for 49 patients and by a phone interview for eight patients who had no possibility for a visit (two living at a remote location, three not willing to attend a visit, two patients not being able to attend a visit due to other serious medical conditions, one patient not speaking Finnish, Swedish nor English interviewed by a translator). Figure 1 presents the flow-chart of study patients. The surviving patients were evaluated six to eight months (median 7.0 months) after the CA. Their clinical and demographical data are presented in Table 1. Diagnostic cardiologic examinations and therapeutic interventions were commonly performed during the initial hospital stay. Coronary angiography had been performed on 51 (89%) patients, percutaneous coronary intervention (PCI) on 21 (37%) patients and coronary artery bypass grafting (CABG) on 7 (12%) patients. Electrophysiological testing had been performed on 17 (30%) patients and an implantable cardioverter defibrillator (ICD) had been implanted in 19 (33%) of the 57 patients.

**Table 1:** **Clinical and demographical data on patients alive six months after out-of-hospital cardiac arrest (N = 57)**

Age, years	59 (24-78)
Male	44 (77%)
Bystander initiated CPR	38 (67%)
BLS, min	8 (2-14)
ACLS, min	14.5 (6-100)
ROSC, min	17 (7-33)
Length of ICU stay, days	7 (3-38)
Serum NSE at 24 hours, mmol/L	16.0 (8.6-41.2)
Serum NSE at 48 hours, mmol/L	14.8 (6.8-33.1)
Delay to recovery of consciousness, days	1 (0-7)
**The aetiology of cardiac arrest**	
Acute myocardial infarction	21 (37%)
Myocardial ischemia without infarction	11 (19%)
Arrhythmia	22 (39%)
Other	3 (5%)*
**Pre-arrest medical history of**	
Coronary heart disease	13 (23%)
Acute myocardial infarction	10 (18%)
Ventricular tachycardia or ventricular fibrillation	2 (4%)
Cardiac insufficiency	11 (19%)
Hypertension	23 (40%)
Diabetes	7 (12%)
Hyperlipidemia, medication prescribed	13 (23%)
Smoker/ex-smoker	18 / 11 (32/19%)

Data are given as absolute numbers (percentage) or as median and range. CPR = cardiopulmonary resuscitation, BLS = basic life support, ACLS = advanced cardiac life support, ROSC = restoration of spontaneous circulation, ICU = intensive care unit, NSE = neuron specific enolase. Delay to recovery of consciousness has been counted from the withdrawal of sedative medication. *Other aetiology of cardiac arrest: unknown for one subject, technical failure of an implantable cardioverter defibrillator in one subject, and myocardial sarcoidosis in one subject.

No difference was observed in the delay to recovery of consciousness, CPC, cognitive outcome, NIHSS, mRs, BI outcome or HRQoL between the strict and moderate glucose groups (data not shown). Therefore we present the outcome data as one group.

At evaluation after CA, CPC 1 outcome had been achieved by 38 patients (42%), CPC 2 by 12 patients (13%), and CPC 3 by 7 patients (8%). None of the patients were in persistent vegetative state (CPC 4), and 33 (37%) had died (CPC 5). Two patients with CPC 2 outcome had already pre-arrest CPC of 2, and post-arrest CPC 3 patients included one patient with a pre-arrest CPC level of 3 and two patients with pre-arrest CPC of 2. Thus, favourable outcome after CA (CPC 1 or 2) was observed in 50 (88% of 57 survivors and 56% of all 90 randomized patients). See Table 2 for additional data on functional outcome.

**Table 2:** **Functional outcome of patients alive six months after cardiac arrest (N = 57)**

**Lives at home**	52 (91%)
Lives with family	46 (81%)
Lives alone	6 (11%)
Receives some help from family members	8 (15%)
Receives some help from social home-care system*	1 (2%)
**Institutional care**	5 (9%)
Sheltered home**	2 (4%)
Nursing home	1 (2%)
Long-term hospital	2 (4%)
**Employed at the time of cardiac arrest**	26 (46%)
Returned to previous employment	16 (61%)
On sick-leave, returned to work later on	3 (12%)
Retired from previous work due to the event	7 (27%)

Data are given as absolute numbers (percentage). *One alone-living patient received help from a home-care nurse once a week with medication dispensing. **These two patients had already lived in a sheltered home before cardiac arrest.

**Figure 2:**
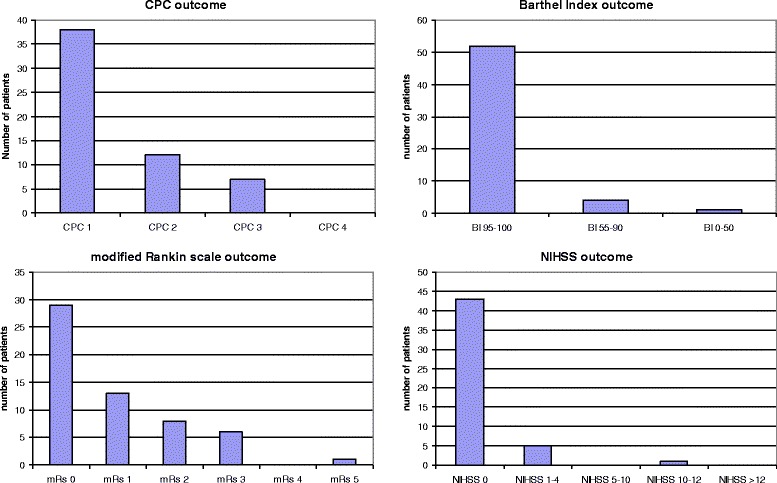
**CPC, Modified Rankin, Barthel Index and NIHSS six months after cardiac arrest.** y-axis shows the percentage of patients.

The distribution of CPC, mRs, BI and NIHSS scores among the survivors are presented as Figure 2. Median [IQR] mRs score was 0 [0-2], median BI score 100 [100-100] and median NIHSS score 0 [0-0]. Of 57 survivors, the outcome was good in 52 (91%) by Barthel Index (BI 95-100), and in 50 (88%) by mRS (mRS 0-2).

Neurological sequels of CA were relatively few. Two patients suffered also an ischemic stroke during the hospitalization for CA, and one of them presented with aphasia and apraxia with NIHSS score of 12. One patient had been diagnosed with post-arrest epilepsy (secondarily generalized seizures) and used antiepileptic medication with good seizure-control. Another patient had experienced focal myoclonic jerks, which had spontaneously declined over time.

Neuropsychological examination could be performed to 41 of the 49 patients attending follow-up visit (72% of all 57 patients alive). Reasons for exclusion were chronic conditions affecting cognitive skills (N = 4; two patients with mental retardation, one patient with chronic schizophrenia and one patient with frontal infarct not able to co-operate), refusal (N = 3) and poor general condition (N = 1). Of 41, 20 (49%) were cognitively intact, 14 (34%) had mild to moderate cognitive deficits and severe cognitive deficits were found in 7 patients (17%). Cognitive deficits were predominantly detected in executive and memory functions.

**Figure 3:**
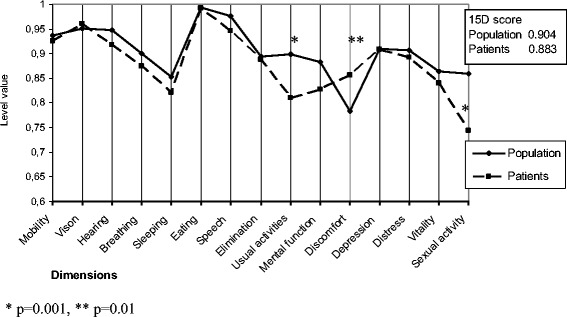
**Self-assessed health-related quality of life by 15D in cardiac arrest survivors at six months compared to age- and gender matched population.**

The 15D profile of studied patients compared to age- and gender matched normal population sample (N = 5689) is presented in Figure 3. The 15D total score of studied patients did not differ from the score of age- and gender matched general population (0.883 vs 0.904, p = 0.112). The scores for two dimensions, usual activities and sexual activities, were significantly lower, whereas the score for one dimension, discomfort and symptoms, was significantly better than the respective scores in the general population sample matched with the gender and age distribution of the patients. Both mRs and CPC scores and classification by cognition correlated with self-assessed 15D total score (for CPC r = -0.425, for mRs r = -0.574, for cognition r = -0.317, p < 0.05). The 15D total score differed significantly between patients with mRs 0 and 1 (median score 0.952 vs 0.851, p = 0.012), between mRs 0 and 2 (median score 0.952 vs 0.730, p = 0.003) and between 0 and 1-2 (median score 0.952 vs 0.840, p < 0.001). The 15D total score difference was also significant between patients with CPC 1 and 2 (median score 0.939 vs 0.824, p = 0.017). However, the 15D total score did not differ between cognitively intact subjects and those with mild to moderate cognitive deficits (0.952 vs 0.885, p = 0.323).

## Discussion

We found that 91% of OHCA-VF patients surviving six months after CA were functionally independent and 95% of survivors had been able to return to their home. Furthermore, the HRQoL of CA survivors did not differ from that of age- and sex-matched normal population.

The proportion of independent patients in this study was higher than the previously reported 65% one year after CA from the pre-hypothermia era [24], and comparable to that of hypothermia-treated patients reported by Cronberg et al [25]. Focal neurological sensomotor deficits among CA survivors are relatively scarce, which was also reflected in the low NIHSS scores among our study patients. The CPC outcome in our study was comparable to that of our Hypothermia After Cardiac Arrest -study patients [26]. In resuscitation studies it is usually assumed that all CA patients have a pre-arrest CPC level of 1, which is not the case in clinical settings. Thus, measuring change from assumed pre-arrest CPC level in addition to the achieved CPC level could reflect more accurately the clinical outcome.

Recent large prospective multicenter study reported that in medical and surgical ICU patients one out of four patients had cognitive impairment 12 months after critical illness, similar in severity to that of patients with mild Alzheimer’s disease; and that neurocognitive dysfunction occurred both in young and old patients [27]. Neuropsychological deficits are common in survivors of CA, ranging from mild deficits in memory and executive functions to severe amnestic syndrome. In the present study half of studied survivors were cognitively intact at six months after CA. As in previous studies with detailed cognitive testing [9,25,28], the most frequent cognitive deficits were found in memory and executive functions. Extensive neuropsychological testing is not routinely offered for all CA survivors, and studies concentrating on predictors of cognitive outcome would be of great importance in order to detect survivors needing further cognitive evaluation and rehabilitation. Mild or moderate cognitive impairments do not necessarily translate to deficits in the activities in the daily living or threaten independency, but especially in the current demanding work life even subtle cognitive deficits may severely impair the person’s working capacity. Recognition of cognitive defects would thus be important, as awareness of limitations enables the use of compensating strategies. In our study 73% of survivors employed at the time of CA returned to work. This high percentage could be related to the relatively small number of patients, but in previous studies this number has also been quite high, between 42-79% [25,29-31].

In the study of Hsu et al, the CPC score at hospital discharge correlated poorly with all categories of the QoL measurements performed at least 6 months later [32]. In our study the HRQoL score correlated well with CPC and mRs assessed by a neurologist at six-months after CA. Of note, for both CPC and mRs, there were significant differences in HRQoL between outcome classes generally regarded as good outcome (CPC 1-2 and mRs 0-2). It seems that even mild residual symptoms are reflected as lower self-assessed QoL. On the other hand, mild to moderate cognitive deficits did not result in significantly lower self-assessed HRQoL. A possible explanation for this is that 15D emphasizes physical symptoms, compared to cognitive complaints. It is also possible that patients with mild cognitive decline manage quite well in familiar environment, adapt to their deficits, or alternatively are unaware of their cognitive deficits. Further studies examining the association of cognition and quality of life using methods sensitive to symptoms caused by cognitive deficits would be of importance.

Previous studies have suggested that most survivors of out-of-hospital CA present a satisfactory quality of life comparable to that of age- and disease-matched controls [33-38]. In the study of Cronberg et al, CA survivors had a slightly lower HRQoL measured by the EQ-5D Visual Analogue Scale than an age-adjusted healthy cohort [25]. In our study the HRQoL of CA survivors did not differ from that of age- and sex-matched control population. In fact, their overall HRQoL measured by the 15D single index score (median 0.883) was higher than previously published scores for patients with coronary artery disease six months after CABG (mean 0.858, SD 0.110) or PCI (mean 0.847, SD 0.105) [21] and higher than after stroke (median 0.86 [22], mean 0.801 [23]).

This study has some important limitations. First, 28% of patients did not attend the neuropsychological examination. It is possible that subjects with cognitive deficits were more prone to refuse the neuropsychological examination which may have caused bias. Second, we cannot exclude the possibility that some of the noticed cognitive deficits existed already before CA, although we tried to exclude patients with pre-existing major cognitive impairment. Third, due to the strict inclusion and exclusion criteria, the results cannot be generalized to all OHCA patients treated in the ICU with hypothermia. Finally, the number of patients was inadequate to find any differences between standard and intensive glucose control, if present. Therefore, we analyzed the patients as one group.

## Conclusions

In this study the functional outcome six months after CA and therapeutic hypothermia was good (CPC1-2) in 88% of the survivors, and half of them were cognitively intact. 95% of survivors had been able to return to their home, and 73% of patients employed at the time of CA had returned to work. Those who survived to six months after CA had quality of life comparable to age- and gender-matched population.
